# Supplementary material for: Decoding necrosome assembly: harmonizing signal amplification and attenuation through optimal RIP3 stoichiometry
Source: Nat Commun. 2025 Dec 23;17:405. doi: 10.1038/s41467-025-67098-5 (PMC12796345; doi:10.1038/s41467-025-67098-5)
Supplement: Supplementary file 1 — Supplementary Information [file 41467_2025_67098_MOESM1_ESM.pdf]

Supplementary Information for

Decoding necrosome assembly: harmonizing signal amplification and attenuation through optimal RIP3 stoichiometry

Xiang Li[1]#*, Yating Cao[2]#, Fei Xu[3]#, Yiting Zhang[2], Yue Kong[2], Chengjie Lan[2], Rongfeng Zhu[4], Cheng Lin[1], Chuan-Qi Zhong[2], Zhilong Liu[1], Hong Qi[5], Yichuan Huang[1], Yunshan Xiao[6], Gui-Quan Sun[5,7], Jianwei Shuai[8]*, Xin Chen[2,9]*;

[1]*Department of Physics, Fujian Provincial Key Lab for Soft Functional Materials Research, Xiamen University, Xiamen 361005, China.*

[2]*State Key Laboratory of Cellular Stress Biology, Innovation Center for Cell Biology, School of Life Sciences, Faculty of Medicine and Life Sciences, Xiamen University, Xiamen 361102, China.*

[3]*Department of Physics, Anhui Normal University, Wuhu 241002, China.*

[4]*Institute of Chemical Biology, Shenzhen Bay Laboratory, Shenzhen 518055, China.*

[5]*Complex Systems Research Center, Shanxi University, Taiyuan 030006, China.*

[6]*Department of Obstetrics, School of Medicine, Xiamen University, Xiamen 361005, China.*

[7]*School of Mathematics, North University of China, Shanxi, Taiyuan 030051, China.*

[8]*Oujiang Laboratory (Zhejiang Lab for Regenerative Medicine, Vision and Brain Health), Wenzhou Institute, University of Chinese Academy of Sciences, Wenzhou 325001, China.*

[9]*Xiamen Cardiovascular Hospital of Xiamen University, School of Medicine, Fujian Branch of National Clinical Research Center for Cardiovascular Diseases, Xiamen 361011, China.*

#These authors contributed equally to this work.

**Corresponding author. Email: xianglibp@xmu.edu.cn (X.L.); shuaijw@wiucas.ac.cn (J.S.); xchen@xmu.edu.cn (X.C.)*

Content:

Supplementary Figures 1 to 7

Supplementary Tables 1 to 2

Supplementary References

Supplementary Figures

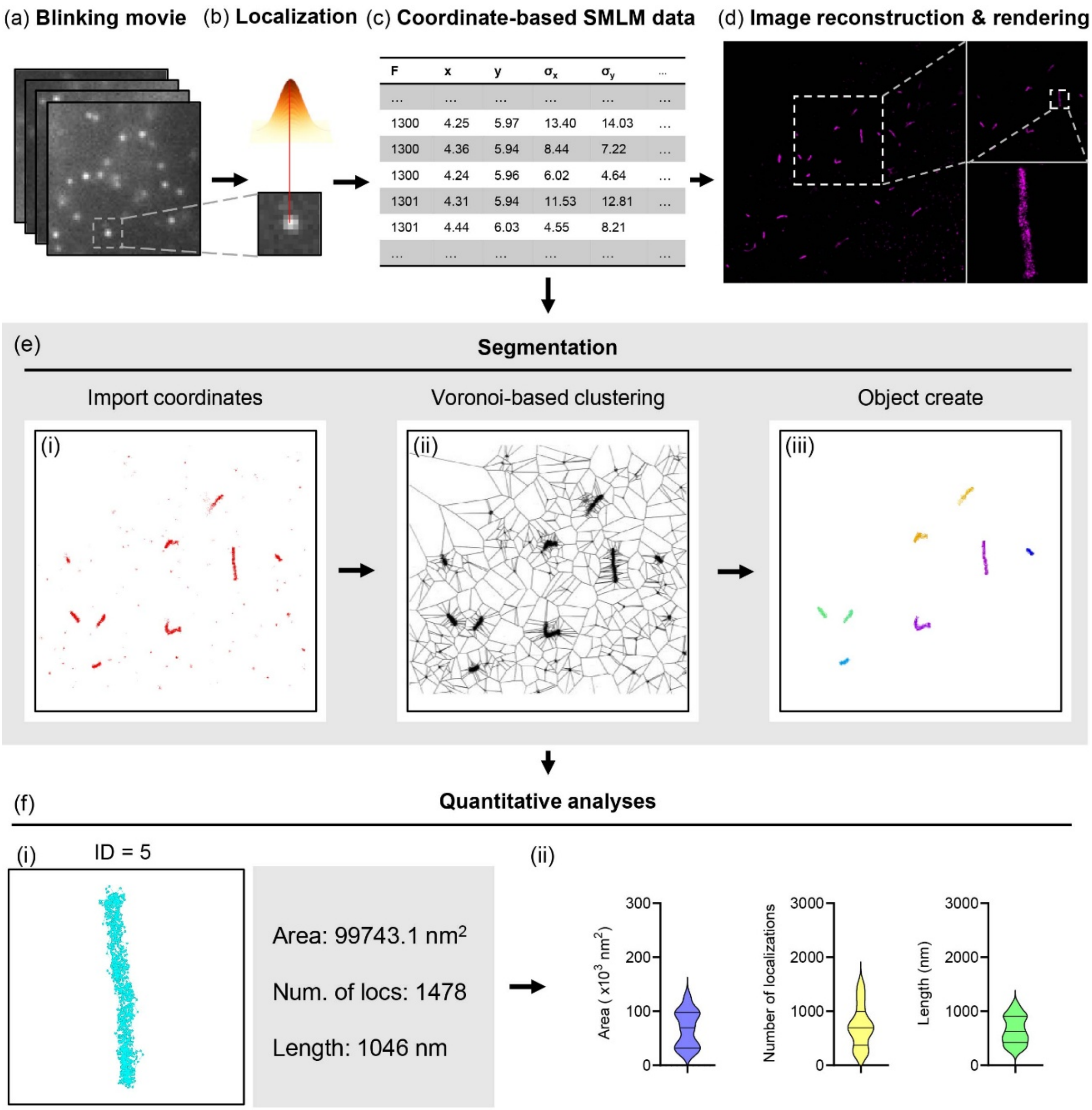

Supplementary Figure 1 | Workflow for single-color STORM image analysis.

a–f Schematic of the analytical pipeline for processing and quantifying single-color STORM images. STORM datasets were acquired as previously described [1]. The procedure involved image acquisition (**a**), single-molecule localization (**b**), coordinate extraction (**c**), and image reconstruction and rendering (**d**). Localization data were then processed using Voronoi-based clustering (**e**). The area and total localization count of clusters were extracted using POCA software, and cluster length was quantified by calculating the maximum Feret diameter with a custom Python script (**f**). All statistical analyses were based on processed localization data. For further details, refer to the Methods section.

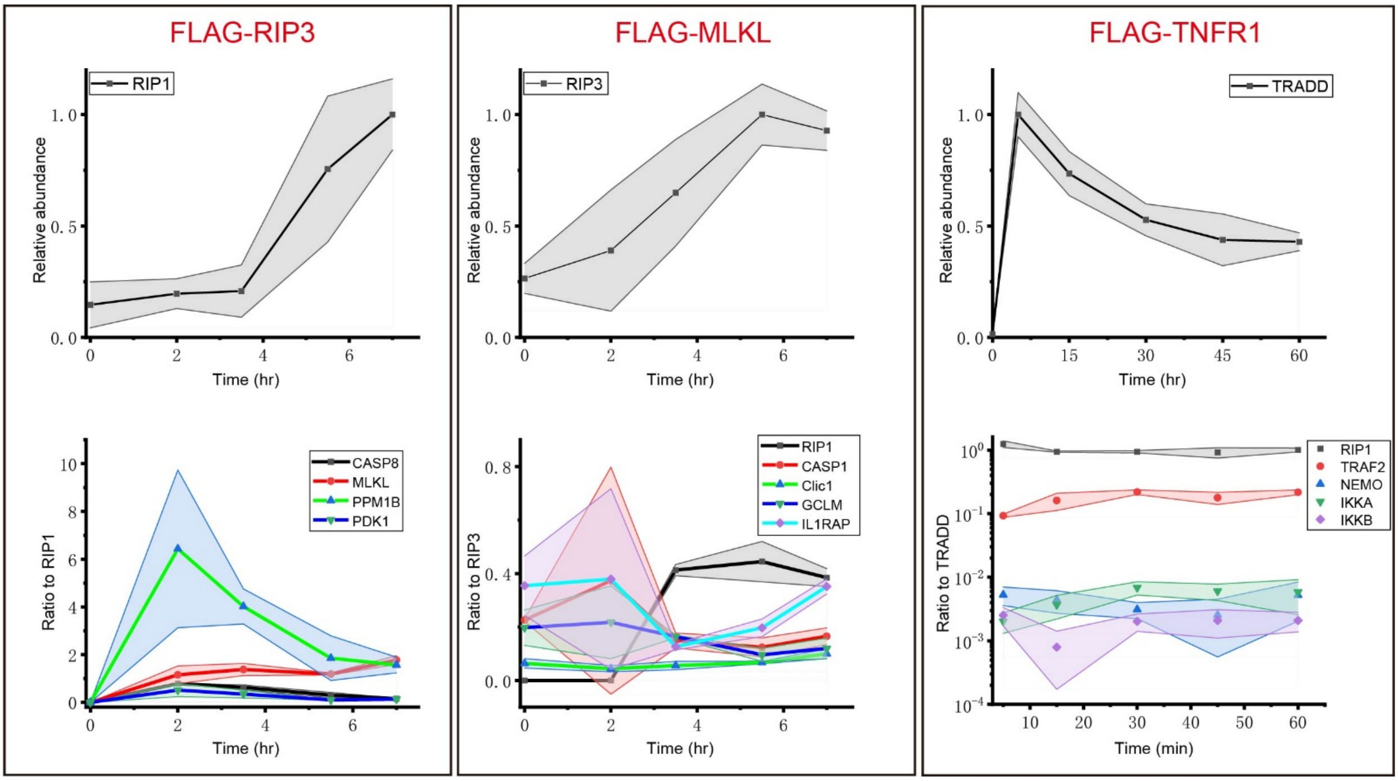

Supplementary Figure 2 | Changes in core protein levels within complexes detected by mass spectrometry.

Temporal variations in the amounts of core proteins, specifically RIP3 and MLKL, within necrosomes and TNFR1 complex are illustrated. The upper panel displays the normalized trends for RIP1, RIP3, and TRADD across different complexes over time, while the lower panel highlights the changes in the proportional relationships of these proteins. Data are presented as mean ± SD from three biological replicates per group. Source data are provided as a Source Data file.

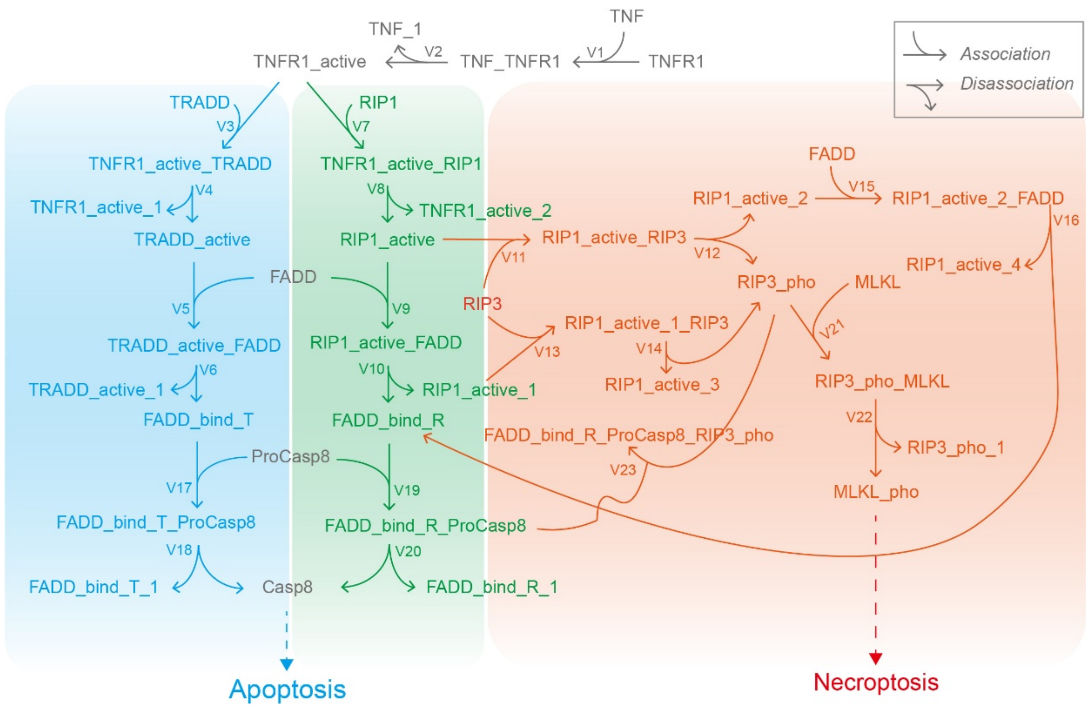

Supplementary Figure 3 | Kinetic scheme of the TNF-induced cell death signaling.

The signaling pathway comprises three main modules: the TRADD-dependent apoptosis module (blue background), the RIP1-dependent apoptosis module (green background), and the RIP3-dependent necroptosis module (orange background). Lines terminating with specific symbols represent chemical reactions characterized by reactions V1-V22, as detailed in Supplementary Table 2. Based on this reaction signaling network, we developed mathematical models that incorporate protein interactions, phosphorylation, and enzymatic reactions. These models are formulated as a set of coupled ordinary differential equations (ODEs), describing the temporal evolution of component concentrations within complexes. The models illustrated in Figs. 3d and 4f, which describe necrosome formation, primarily encompass the reactions depicted in the green and orange backgrounds, whereas the models in Fig. 7a involve reactions represented in the green and blue backgrounds, respectively.

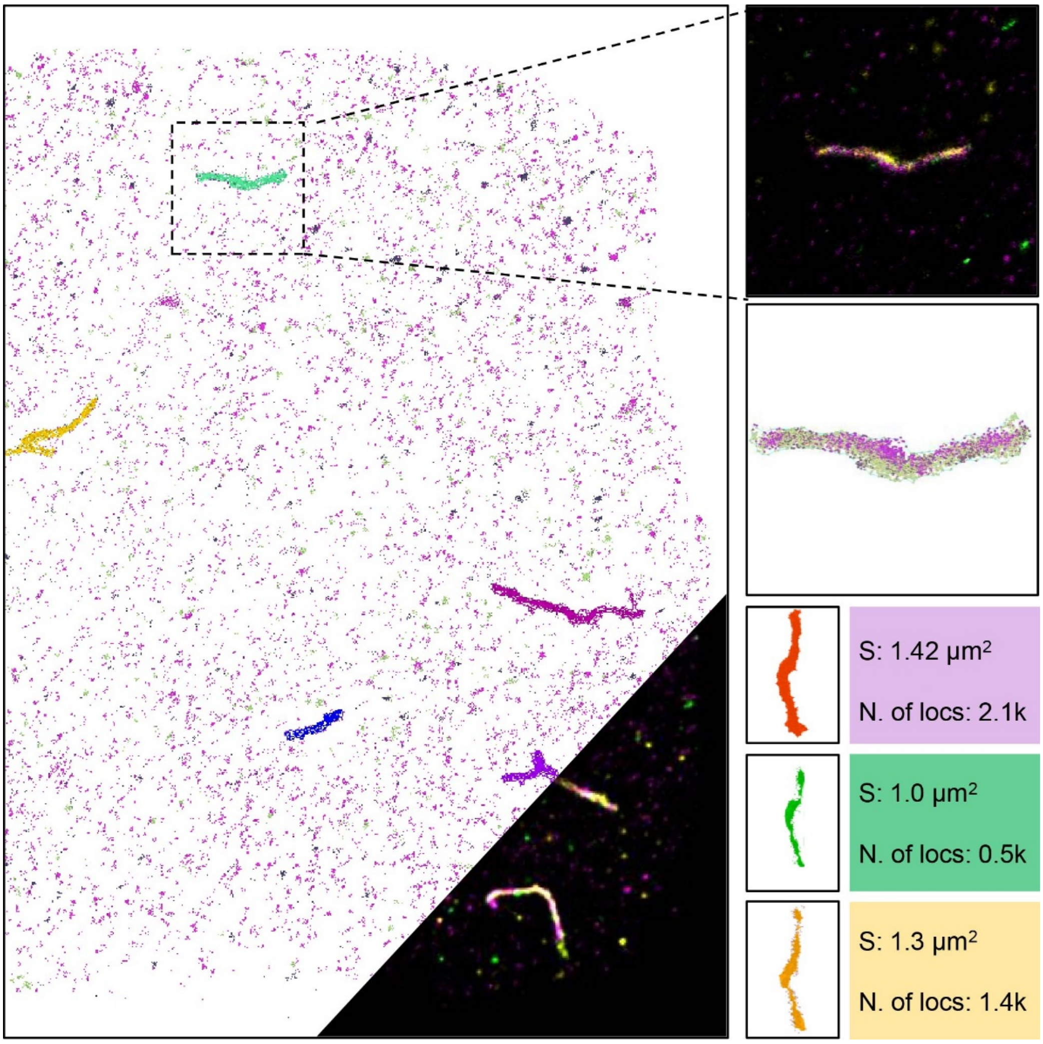

Supplementary Figure 4 | Workflow for multi-color STORM image analysis.

Clusters in individual channels (RIP1: green; RIP3: red; p-RIP3: yellow) were segmented using POCA, followed by intra- and inter-channel overlap analysis. For each overlapping cluster, area and localization counts were quantified, enabling calculation of RIP1:RIP3 stoichiometry and relative p-RIP3 levels within individual necrosomes. Detailed methods are described in the Methods section.

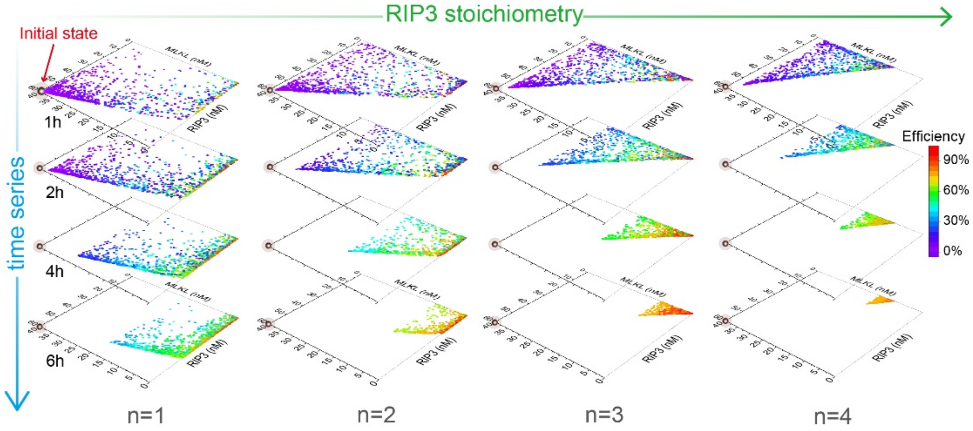

Supplementary Figure 5 | Kinetic evolution paths of different RIP3 stoichiometries under random initial conditions. The horizontal axis depicts various stoichiometries of RIP3, while the vertical axis illustrates the kinetic evolution of the model, initiated from 10,000 random initial conditions. Each point on the plot represents the model state corresponding to a specific initial condition, with color coding indicating the associated phosphorylation efficiency.

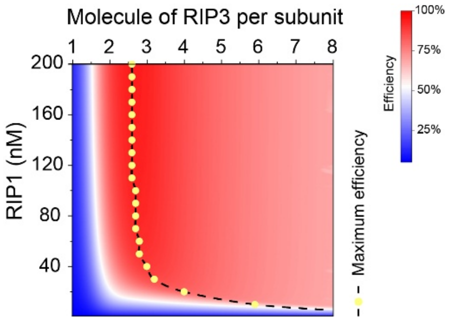

Supplementary Figure 6 | Regulatory effect of RIP1 on RIP3 stoichiometry.

The maximum efficiency indicates the optimal degree of RIP3 assembly achieved under specific conditions.

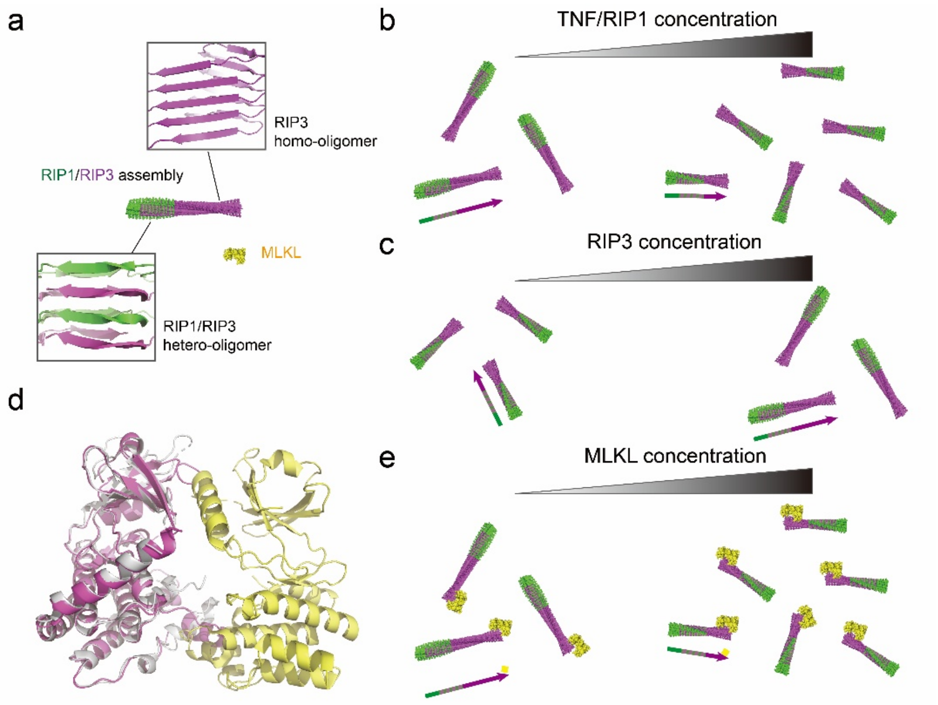

Supplementary Figure 7 | Structural basis for the phase diagram of necrosome assembly.

a Structural models of RIP1–RIP3 hetero-amyloid (PDB: 5V7Z) and RIP3–RIP3 homo-amyloid (PDB: 7DAC) fibrils. **b, c** Proposed impact of varying TNF/RIP1 (**b**) and RIP3 (**c**) levels on necrosome configuration inferred from fibril stoichiometry. **d** Crystal structure of the MLKL-bound RIP3 complex (PDB: 7MON) showing RIP3 in an inactive conformation. **e** Schematic model of RIP3 distribution under high MLKL levels, suggesting MLKL-mediated restriction of RIP3 oligomerization.

Supplementary Tables

Supplementary Table 1 | Basic models of the two and three components assembly.

Reactions and reaction rates of the models.

No.	Reactions	Reaction rates
V1	*m*A+n*B = AB_mn*	$k1*A^{m}*B^{n}$
V2	*AB_mn =m*A_ + n*B_*	*k2*AB_mn*
V3	*n*B_ + h*C = BC_nh*	$k3*B\_^{n}*C^{h}$
V4	*BC_nh =n*B_1+h*C_*	*k4*BC_nh*

Ordinary differential equations (ODEs) and initial amounts.

No.	Components	ODEs	Initial amounts
E1	*A*	*d[A]/dt= -m*V1*	10
E2	*B*	*d[B]/dt= -n*V1*	10
E3	*AB_mn*	*d[AB_mn]/dt= V1-V2*	0
E4	*A_*	*d[A_]/dt= m*V2*	0
E5	*B_*	*d[B_]/dt= n*V2-n*V3*	0
E6	*C*	*d[C]/dt= -h*V3*	10
E7	*BC_nh*	*d[BC_nh]/dt= V3-V4*	0
E8	*B_1*	*d[B_1]/dt= n*V4*	0
E9	*C_*	*d[C_]/dt= h*V4*	0

Parameters values and descriptions of the models.

Parameters	Description	References
$k1=10^{-4}$	Rates of A binds to B	[2]
k2=1.0	Activation rate of B in complex	[2]
$k3=10^{-4}$	Rates of AB complex binds to C	[2]
k4=1.0	Activation rate of C in complex	[2]
m=1~8	Assembly degree of A in complex	
n=1~8	Assembly degree of B in complex	
h=1~8	Assembly degree of C in complex	

Supplementary Table 2 | Mathematical modeling of the TNF-induced cell death signaling.

Reactions and reaction rates of the models.

No.	Reactions	Reaction rates
V1	TNF+TNFR1 = TNF_TNFR1	k1*TNF*TNFR1-k_1*TNF_TNFR1
V2	TNF_TNFR1 =TNF_1 + TNFR1_active	k2*TNF_TNFR1
V3	TNFR1_active + TRADD = TNFR1_active_TRADD	k3*TNFR1_active*TRADD-k_3*TNFR1_active_TRADD
V4	TNFR1_active_TRADD=TNFR1_active_1+TRADD_active	k4*TNFR1_active_TRADD
V5	TRADD_active + FADD = TRADD_active_FADD	k5*TRADD_active*FADD-k_5*TRADD_active_FADD
V6	TRADD_active_FADD=TRADD_active_1+ FADD_bind_T	k6*TRADD_active_FADD
V7	TNFR1_active + RIP1 = TNFR1_active_RIP1	k7*TNFR1_active*RIP1-k_7*TNFR1_active_RIP1
V8	TNFR1_active_RIP1 = TNFR1_active_2 + RIP1_active	k8*TNFR1_active_RIP1
V9	RIP1_active + FADD = RIP1_active_FADD	k9*FADD*RIP1_active-k_9*RIP1_active_FADD
V10	RIP1_active_FADD = RIP1_active_1 + FADD_bind_R	k10*RIP1_active_FADD
V11	RIP1_active + 3*RIP3 = RIP1_active_RIP3_3	k11*RIP1_active*RIP3^nRIP3-k_11*RIP1_active_RIP3_3
V12	RIP1_active_RIP3_3 = RIP1_active_2 + 3*RIP3_pho	k12*RIP1_active_RIP3_3
V13	RIP1_active_1 + 3*RIP3 = RIP1_active_1_RIP3_3	k13*RIP1_active_1*RIP3^nRIP3-k_13*RIP1_active_1_RIP3_3
V14	RIP1_active_1_RIP3_3 = RIP1_active_3 + 3*RIP3_pho	k14*RIP1_active_1_RIP3_3
V15	RIP1_active_2 + FADD = RIP1_active_2_FADD	k15*FADD*RIP1_active_2-k_15*RIP1_active_2_FADD
V16	RIP1_active_2_FADD = RIP1_active_4 + FADD_bind_R	k16*RIP1_active_2_FADD
V17	FADD_bind_T + 2*ProCasp8 = FADD_bind_T_ProCasp8_2	k17*FADD_bind_T*ProCasp8^nPC8-k_17*FADD_bind_T_ProCasp8_2
V18	FADD_bind_T_ProCasp8_2 = FADD_bind_T_1 + 2*Casp8	k18*FADD_bind_T_ProCasp8_2
V19	FADD_bind_R + ProCasp8 = FADD_bind_R_ProCasp8	k19*FADD_bind_R*ProCasp8-k_19*FADD_bind_R_ProCasp8
V20	FADD_bind_R_ProCasp8 = FADD_bind_R_1 + Casp8	k20*FADD_bind_R_ProCasp8
V21	3*RIP3_pho + 2*MLKL = RIP3_pho_3_MLKL_2	k21*RIP3_pho^nRIP3*MLKL^nMLKL-k_21*RIP3_pho_3_MLKL_2
V22	RIP3_pho_3_MLKL_2 = 3*RIP3_pho_1 + 2*MLKL_pho	k22*RIP3_pho_3_MLKL_2
V23	FADD_bind_R_ProCasp8 + RIP3_pho = FADD_bind_R_ProCasp8_RIP3_pho	k23*RIP3_pho*FADD_bind_R_ProCasp8-k_23*FADD_bind_R_ProCasp8_RIP3_pho

Supplementary Table 2 (continued) | Mathematical modeling of the TNF-induced cell death signaling.

Ordinary differential equations (ODEs) and initial amounts.

No.	Components	ODEs	Initial amounts (mpc)	References
E1	TNF	d[TNF]/dt= -V1	4×10^4	Estimated
E2	TNFR1	d[TNFR1]/dt= -V1	4×10^4	Estimated
E3	TNF_TNFR1	d[TNF_TNFR1]/dt= V1-V2	0	
E4	TNF_1	d[TNF_1]/dt= V2	0	
E5	TNFR1_active	d[TNFR1_active]/dt= V2-V3-V7	0	
E6	TRADD	d[TRADD]/dt= -V3	4×10^4	Estimated
E7	TNFR1_active_TRADD	d[TNFR1_active_TRADD]/dt= V3-V4	0	
E8	TNFR1_active_1	d[TNFR1_active_1]/dt= V4	0	
E9	TRADD_active	d[TRADD_active]/dt= V4-V5	0	
E10	FADD	d[FADD]/dt= -V5-V9-V15	2.5×10^4	[3]
E11	TRADD_active_FADD	d[TRADD_active_FADD]/dt= V5-V6	0	
E12	TRADD_active_1	d[TRADD_active_1]/dt= V6	0	
E13	FADD_bind_T	d[FADD_bind_T]/dt= V6-V17	0	
E14	RIP1	d[RIP1]/dt= -V7	5×10^4	[4]
E15	TNFR1_active_RIP1	d[TNFR1_active_RIP1]/dt= V7-V8	0	
E16	TNFR1_active_2	d[TNFR1_active_2]/dt= V8	0	
E17	RIP1_active	d[RIP1_active]/dt= V8-V9-V11	0	
E18	RIP1_active_FADD	d[RIP1_active_FADD]/dt= V9-V10	0	
E19	RIP1_active_1	d[RIP1_active_1]/dt= V10-V13	0	
E20	FADD_bind_R	d[FADD_bind_R]/dt= V10+V16-V19	0	
E21	RIP3	d[RIP3]/dt=-V11*nRIP3-V13*nRIP3	3×10^4	[4]
E22	RIP1_active_RIP3_3	d[RIP1_active_RIP3_3]/dt= V11-V12	0	
E23	RIP1_active_2	d[RIP1_active_2]/dt= V12-V15	0	
E24	RIP3_pho	d[RIP3_pho]/dt=V12*nRIP3+V14*nRIP3 -V21*nRIP3-V23	0	
E25	RIP1_active_1_RIP3_3	d[RIP1_active_1_RIP3_3]/dt=V13-V14	0	
E26	RIP1_active_3	d[RIP1_active_3]/dt=V14	0	
E27	RIP1_active_2_FADD	d[RIP1_active_2_FADD]/dt=V15-V16	0	
E28	RIP1_active_4	d[RIP1_active_4]/dt=V16	0	
E29	ProCasp8	d[ProCasp8]/dt=-V17*nPC8-V19	3×10^4	[4]
E30	FADD_bind_T_ProCasp8 _2	d[FADD_bind_T_ProCasp8_2]/dt=V17-V18	0	
E31	FADD_bind_T_1	d[FADD_bind_T_1]/dt=V18	0	
E32	Casp8	d[Casp8]/dt=V18*nPC8+V20	0	
E33	FADD_bind_R_ProCasp8	d[FADD_bind_R_ProCasp8]/dt=V19-V20-V23	0	
E34	FADD_bind_R_1	d[FADD_bind_R_1]/dt=V20	0	
E35	MLKL	d[MLKL]/dt=-V21*nMLKL	2×10^4	[4]
E36	RIP3_pho_3_MLKL_2	d[RIP3_pho_3_MLKL_2]/dt=V21-V22	0	
E37	RIP3_pho_1	d[RIP3_pho_1]/dt=V22*nRIP3	0	
E38	MLKL_pho	d[MLKL_pho]/dt=V22*nMLKL	0	

E39	*FADD_bind_R_ProCasp8_RIP3_pho*	*d[FADD_bind_R_ProCasp8_RIP3_pho]/dt=V23*	0	

Parameters values and descriptions of the models.

Parameters	Description	References
k1=10^{-5}, *k_1*=10^{-3}	Rates of TNF binds to TNFR1	[2]
k2=10^{-3}	Rate of TNFR1_active drops from TNF_TNFR1 complex	[2]
k3=1.5×10^{-6}, *k_3*=10^{-3}	Rates of TNFR1_active binds to TRADD	Fitted and [2]
k4=10^{-3}	Rate of TRADD_active drops from TNFR1_active_TRADD complex	[2]
k5=10^{-8}, *k_5*=10^{-3}	Rates of TNFR1_active binds to FADD	Fitted and [2]
k6=10^{-3}	Rate of FADD_bind_T drops from TRADD_active_FADD complex	[2]
k7=10^{-6}, *k_7*=10^{-3}	Rates of TNFR1_active binds to RIPI	Fitted and [2]
k8=10^{-3}	Rate of RIP1_active drops from TNFR1_active_RIP1 complex	[2]
k9=10^{-6}, *k_9=1.0e-3*	Rates of RIP1_active binds to FADD	Fitted and [2]
k10=10^{-3}	Rate of FADD_bind_R drops from RIP1_active_FADD complex	[2]
k11=10^{-14}, *k_11*=10^{-3}	Rates of RIP1_active binds to RIP3	Fitted and [2]
k12=10^{-3}	Rate of RIP3_pho drops from RIP1_active_RIP3_3	[2]
k13=10^{-14}, *k_13*=10^{-3}	Rates of RIP1_active_1 binds to RIP3	Fitted and [2]
K14=10^{-3}	Rate of RIP3_pho drops from RIP1_active_RIP3_3	[2]
k15=10^{-6}, *k_15*=10^{-3}	Rates of RIP1_active_2 binds to FADD	Fitted and [2]
k16=10^{-3}	Rate of FADD_bind_R drops from RIP1_active_2_FADD	[2]
k17=10^{-6}, *k_17*=10^{-3}	Rates of FADD_bind_T binds to ProCasp8	Fitted and [2]
k18=10^{-5}	Rate of Casp8 drops from FADD_bind_T_ProCasp8_2	Fitted
k19=10^{-6}, *k_19*=10^{-3}	Rates of FADD_bind_R binds to ProCasp8	Fitted and [2]
k20=10^{-5}	Rate of Casp8 drops from FADD_bind_R_ProCasp8	Fitted
k21=10^{-12}, *k_21*=10^{-3}	Rates of RIP3_pho binds to MLKL	Fitted and [2]
k22=10^{-3}	Rate of MLKL_pho drops from RIP3_pho_3_MLKL_2	[2]
k23=10^{-2}, *k_23*=10^{-2}	Rates of FADD_bind_R_ProCasp8 binds to RIP3_pho	[2]

Supplementary References

[1] Chen X, Zhu R, Zhong J, et al. Mosaic composition of RIP1-RIP3 signalling hub and its role in regulating cell death. Nat Cell Biol. 2022;24(4):471-482.

[2] Albeck J G, Burke J M, Spencer S L, et al. Modeling a snap-action, variable-delay switch controlling extrinsic cell death. PLoS biology. 2008;6(12):e299.

[3] Schwanhäusser B, Busse D, Li N, et al. Global quantification of mammalian gene expression control. Nature. 2011;473(7347):337-342.

[4] Li X, Zhong C Q, Wu R, et al. RIP1-dependent linear and nonlinear recruitments of caspase-8 and RIP3 respectively to necrosome specify distinct cell death outcomes. Protein & Cell. 2021;12(11):858-876.
