# Supplementary material for: Decoding necrosome assembly: harmonizing signal amplification and attenuation through optimal RIP3 stoichiometry
Source: Nat Commun. 2025 Dec 23;17:405. doi: 10.1038/s41467-025-67098-5 (PMC12796345; doi:10.1038/s41467-025-67098-5)
Supplement: Supplementary file 2 — Description of Additional Supplementary Files [file 41467_2025_67098_MOESM2_ESM.pdf]

Description of Additional Supplementary Files

Supplementary Data 1. Sources and identifiers of reagents.

Supplementary Data 2. Details of antibodies used in this study.
