# Supplementary material for: Decoding necrosome assembly: harmonizing signal amplification and attenuation through optimal RIP3 stoichiometry
Source: Nat Commun. 2025 Dec 23;17:405. doi: 10.1038/s41467-025-67098-5 (PMC12796345; doi:10.1038/s41467-025-67098-5)
Supplement: Supplementary file 5 — Reporting Summary [file 41467_2025_67098_MOESM5_ESM.pdf]

nature portfolio

Corresponding author(s): Liang Li, Jianwei Shuai, Xin Chen

Last updated by author(s): Oct 26, 2025

Reporting Summary

Nature Portfolio wishes to improve the reproducibility of the work that we publish. This form provides structure for consistency and transparency in reporting. For further information on Nature Portfolio policies, see our Editorial Policies and the Editorial Policy Checklist.

Statistics

For all statistical analyses, confirm that the following items are present in the figure legend, table legend, main text, or Methods section.

n/a	Confirmed	
☐	☒	The exact sample size (*n*) for each experimental group/condition, given as a discrete number and unit of measurement
☐	☒	A statement on whether measurements were taken from distinct samples or whether the same sample was measured repeatedly
☐	☒	The statistical test(s) used AND whether they are one- or two-sided<br>*Only common tests should be described solely by name; describe more complex techniques in the Methods section.*
☒	☐	A description of all covariates tested
☒	☐	A description of any assumptions or corrections, such as tests of normality and adjustment for multiple comparisons
☐	☒	A full description of the statistical parameters including central tendency (e.g. means) or other basic estimates (e.g. regression coefficient) AND variation (e.g. standard deviation) or associated estimates of uncertainty (e.g. confidence intervals)
☐	☒	For null hypothesis testing, the test statistic (e.g. *F*, *t*, *r*) with confidence intervals, effect sizes, degrees of freedom and *P* value noted<br>*Give P values as exact values whenever suitable.*
☒	☐	For Bayesian analysis, information on the choice of priors and Markov chain Monte Carlo settings
☒	☐	For hierarchical and complex designs, identification of the appropriate level for tests and full reporting of outcomes
☒	☐	Estimates of effect sizes (e.g. Cohen's *d*, Pearson's *r*), indicating how they were calculated

Our web collection on statistics for biologists contains articles on many of the points above.

Software and code

Policy information about availability of computer code

Data collection	All the instruments used are commercially available and were controlled using the software by the manufacturer.<br>AI 680 software (GE Healthcare, Amersham Imager 680)<br>ZEN 2019 v. 3.3.89.0006 (Zeiss, LSM980 confocal microscopy)<br>NIS-Elements AR software with N-STORM module v. 4.50.00 (Nikon, N-STORM super-resolution microscopy)<br>Analysis (SCIEX, TripleTOF 5600 mass spectrometry)
Data analysis	SMAP (for reconstruction, chromatic calibration, and visualization of super-resolution images)<br>POCA (for cluster segmentation of STORM images)<br>NIS-Elements AR with N-STORM module (Nikon, for reconstruction, chromatic calibration, and visualization of super-resolution images)<br>Fiji/Image J v. 1.53f51 (for Feret parameter and area extraction; data visualization)<br>Excel 2016 (Microsoft, for image analysis data management)<br>Prism v. 9.5 (GraphPad, for statistical analysis and plotting)<br>DIA-NN v. 1.8.1 (for analyzing proteomic data)<br>Custom Python code for STORM image analysis (for calculating the maximum Feret diameter of POCA-defined clusters, deposited to Github (https://github.com/xchenxmu/POCA_caculation_MAX_Feret_diameter.git))

For manuscripts utilizing custom algorithms or software that are central to the research but not yet described in published literature, software must be made available to editors and reviewers. We strongly encourage code deposition in a community repository (e.g. GitHub). See the Nature Portfolio guidelines for submitting code & software for further information.

Data

Policy information about availability of data

All manuscripts must include a data availability statement. This statement should provide the following information, where applicable:

- Accession codes, unique identifiers, or web links for publicly available datasets
- A description of any restrictions on data availability
- For clinical datasets or third party data, please ensure that the statement adheres to our policy

All data supporting the findings of this study are available within the paper and its Supplementary Information. The proteomic raw data have been deposited to the ProteomeXchange Consortium via the PRIDE repository with the dataset identifier PXD067729. Source data are provided with this paper.

Research involving human participants, their data, or biological material

Policy information about studies with human participants or human data. See also policy information about sex, gender (identity/presentation), and sexual orientation and race, ethnicity and racism.

Reporting on sex and gender	N/A
Reporting on race, ethnicity, or other socially relevant groupings	N/A
Population characteristics	N/A
Recruitment	N/A
Ethics oversight	N/A

Note that full information on the approval of the study protocol must also be provided in the manuscript.

Field-specific reporting

Please select the one below that is the best fit for your research. If you are not sure, read the appropriate sections before making your selection.

☒ Life sciences ☐ Behavioural & social sciences ☐ Ecological, evolutionary & environmental sciences

For a reference copy of the document with all sections, see nature.com/documents/nr-reporting-summary-flat.pdf

Life sciences study design

All studies must disclose on these points even when the disclosure is negative.

Sample size	No statistical methods were used to predetermine sample size, as this study did not include animal models or human participants. Sample size was determined based on standards in the field (Zhou et al., Science, 2019; Shin et al., Nature Communications, 2025; Zaza et al., Nature Communications, 2025) and experimental experience to obtain statistical significance and reproducibility.
Data exclusions	No data were excluded.
Replication	All experimental findings were reliably reproduced in two-four independent experiments as indicated in the figure legends. All attempts at replication were successful.
Randomization	No randomization was done, because this study does not involve animals or human participants. Samples were organized into groups based on treatments (e.g. experimental time-points). Appropriate controls were included in all experiments.
Blinding	For microscopy data collection, researchers were not blinded to samples but fields of view were chosen on a random basis, preventing potentially biased selection for desired phenotypes.

Reporting for specific materials, systems and methods

We require information from authors about some types of materials, experimental systems and methods used in many studies. Here, indicate whether each material, system or method listed is relevant to your study. If you are not sure if a list item applies to your research, read the appropriate section before selecting a response.

Materials & experimental systems

n/a	Involved in the study
☐	☒ Antibodies
☐	☒ Eukaryotic cell lines
☒	☐ Palaeontology and archaeology
☒	☐ Animals and other organisms
☒	☐ Clinical data
☒	☐ Dual use research of concern
☒	☐ Plants

Methods

n/a	Involved in the study
☒	☐ ChIP-seq
☒	☐ Flow cytometry
☒	☐ MRI-based neuroimaging

Antibodies

Antibodies used

The following primary antibodies were used:
-RIP (D94C12) XP® Rabbit mAb (Cell Signaling, Cat#3493)
-Phospho-RIP (Ser166) (D1L3S) Rabbit mAb (Cell Signaling, Cat#65746)
-RIP3 (E1Z1D) Rabbit mAb (Cell Signaling, Cat#13526)
-Anti-RIP3 (phospho S227) antibody [EPR9627] (Abcam, Cat#ab209384)
-Anti-MLKL antibody [EPR17514] (Abcam, Cat#ab184718)
-Anti-MLKL (phospho S358) antibody [EPR9514] (Abcam, Cat#ab187091)
-HA-Tag Antibody (F-7) (Santa Cruz, Cat#sc-7392)
-DYKDDDDK-Tag(3B9) mAb (Abmart, Cat#M20008L)
-GAPDH Monoclonal antibody (Proteintech, Cat#60004-1-Ig)

The following secondary antibodies were used:
-Goat anti-Rabbit IgG (H+L) Highly Cross-Adsorbed Secondary Antibody, Alexa Fluor™ 488 (Thermo Fisher Scientific, Cat#A11034)
-Goat anti-Mouse IgG (H+L) Cross-Adsorbed Secondary Antibody, Alexa Fluor™ 568 (Thermo Fisher Scientific, Cat#A11004)
-Goat anti-Rat IgG (H+L) Cross-Adsorbed Secondary Antibody, Alexa Fluor™ 647 (Thermo Fisher Scientific, Cat#A21247)
-CF®488A Donkey Anti-Rabbit IgG (H+L), Highly Cross-Adsorbed (Biotium, Cat#20015)
-CF®568 Goat Anti-Mouse IgG (H+L), Highly Cross-Adsorbed, CF® Dye Conjugates, Single Label for STORM (Biotium, Cat#20800)
-CF®568 Goat Anti-Rabbit IgG (H+L), Highly Cross-Adsorbed, CF® Dye Conjugates, Single Label for STORM (Biotium, Cat#20801)
-CF®647 Goat Anti-Mouse IgG (H+L), Highly Cross-Adsorbed (Biotium, Cat#20808)
-CF®647 Goat Anti-Mouse IgG (H+L), Highly Cross-Adsorbed, CF® Dye Conjugates, Single Label for STORM (Biotium, Cat#20809)

Validation

All the antibodies used in this study are commercially available, validated by the manufactures or by our laboratory:
-Validation 3493: https://www.cellsignal.com/products/primary-antibodies/rip-d94c12-xp-rabbit-mab/3493
-Validation 65746: https://www.cellsignal.com/products/primary-antibodies/phospho-rip-ser166-d1l3s-rabbit-mab/65746
-Validation 13526: https://www.cellsignal.com/products/primary-antibodies/rip3-e1z1d-rabbit-mab/13526
-Validation ab209384: https://www.abcam.com/en-us/products/primary-antibodies/rip3-phospho-s227-antibody-epr9627-ab209384
-Validation ab184718: https://www.abcam.com/en-us/products/primary-antibodies/mlkl-antibody-epr17514-ab184718
-Validation ab187091: https://www.abcam.com/en-us/products/primary-antibodies/mlkl-phospho-s358-antibody-epr9514-ab187091
-Validation sc-7392: https://www.scbt.com/p/ha-probe-antibody-f-7
-Validation M20008L): https://www.ab-mart.com.cn/page.aspx?node=%2059%20&id=%20968
-Validation 60004-1-Ig: https://www.ptgcn.com/products/GAPDH-Antibody-60004-1-PBS.htm
-Validation A11034: https://www.thermofisher.cn/cn/zh/antibody/product/Goat-anti-Rabbit-IgG-H-L-Highly-Cross-Adsorbed-Secondary-Antibody-Polyclonal/A-11034
-Validation A11004: https://www.thermofisher.cn/cn/zh/antibody/product/Goat-anti-Mouse-IgG-H-L-Cross-Adsorbed-Secondary-Antibody-Polyclonal/A-11004?adobe_mc=MCMID%7C59572573736099078851773797104666050668%7CMCAID%3D337DA7D8A35C59AF-40001D84007EF14F%7CMCORGID%3D5B135A0C5370E6B40A490D44%40AdobeOrg%7CTS=1614293705
-Validation A21247: https://www.thermofisher.cn/cn/zh/antibody/product/Goat-anti-Rat-IgG-H-L-Cross-Adsorbed-Secondary-Antibody-Polyclonal/A-21247?adobe_mc=MCMID%7C59572573736099078851773797104666050668%7CMCAID%3D337DA7D8A35C59AF-40001D84007EF14F%7CMCORGID%3D5B135A0C5370E6B40A490D44%40AdobeOrg%7CTS=1614293705
-Validation 20015: https://biotium.com/product/donkey-anti-rabbit-igg-hl-highly-cross-adsorbed/?attribute_pa_conjugation=cf488a
-Validation 20302: https://biotium.com/product/goat-anti-mouse-igg-hl-highly-cross-adsorbed-min-x-rat/
-Validation 20800: https://biotium.com/product/goat-anti-mouse-igg-hl-highly-cross-absorbed-cf-dye-STORM/
-Validation 20801: https://biotium.com/product/goat-anti-rabbit-igg-hl-highly-cross-absorbed-cf-dye-STORM/
-Validation 20808: https://biotium.com/product/goat-anti-mouse-igg-hl-highly-cross-absorbed/
-Validation 20809: https://biotium.com/product/goat-anti-mouse-igg-hl-highly-cross-absorbed-cf-dye-STORM/

Eukaryotic cell lines

Policy information about cell lines and Sex and Gender in Research

Cell line source(s): HeLa (ATCC-CCL2), HEK293T (ATCC-CRL-3216), and L929 (ATCC-CCL1).

Authentication: No additional authentication was performed.

Mycoplasma contamination: Cells were regularly tested for mycoplasma contamination and were not positive for mycoplasma.

Commonly misidentified lines (See ICLAC register)	No cell lines used in this study were found in the database of commonly misidentified cell lines.

Plants

Seed stocks	N/A
Novel plant genotypes	N/A
Authentication	N/A
